# Pathomechanisms of Paclitaxel-Induced Peripheral Neuropathy

**DOI:** 10.3390/toxics9100229

**Published:** 2021-09-22

**Authors:** Ines Klein, Helmar C. Lehmann

**Affiliations:** 1 Department of Neurology, University Hospital of Cologne, 50937 Cologne, Germany; ines.muke@uk-koeln.de; 2 Center for Molecular Medicine Cologne (CMMC), University of Cologne, 50937 Cologne, Germany

**Keywords:** paclitaxel, CIPN, paclitaxel-induced peripheral neuropathy, neurotoxicity, neuropathy, taxol

## Abstract

Peripheral neuropathy is one of the most common side effects of chemotherapy, affecting up to 60% of all cancer patients receiving chemotherapy. Moreover, paclitaxel induces neuropathy in up to 97% of all gynecological and urological cancer patients. In cancer cells, paclitaxel induces cell death via microtubule stabilization interrupting cell mitosis. However, paclitaxel also affects cells of the central and peripheral nervous system. The main symptoms are pain and numbness in hands and feet due to paclitaxel accumulation in the dorsal root ganglia. This review describes in detail the pathomechanisms of paclitaxel in the peripheral nervous system. Symptoms occur due to a length-dependent axonal sensory neuropathy, where axons are symmetrically damaged and die back. Due to microtubule stabilization, axonal transport is disrupted, leading to ATP undersupply and oxidative stress. Moreover, mitochondria morphology is altered during paclitaxel treatment. A key player in pain sensation and axonal damage is the paclitaxel-induced inflammation in the spinal cord as well as the dorsal root ganglia. An increased expression of chemokines and cytokines such as IL-1β, IL-8, and TNF-α, but also CXCR4, RAGE, CXCL1, CXCL12, CX3CL1, and C3 promote glial activation and accumulation, and pain sensation. These findings are further elucidated in this review.

## 1. Introduction

Cancer patients who receive anticancer treatment develop peripheral neuropathy in up to 60% of cases [1,2,3]. Several cytotoxic agents such as epothilones (ixabepilone), platinum compounds (cisplatin, carboplatin, and oxaliplatin), proteasome inhibitors (bortezomib), taxanes (paclitaxel and docetaxel), vinca alkaloids (vincristine and vinblastine), and immunomodulatory drugs (thalidomide) are able to induce a peripheral neuropathy (CIPN) [4,5,6,7]. The occurrence of pain in hands and feet in a “glove and stocking” pattern is one of the most common dose-limiting factors in treatment [8,9]. Besides pain, CIPN presents with numbness, loss of vibration sense due to the affection of large caliber sensory neurons, as well as dysesthesia and cold and mechanical hypersensitivity, functions that are executed by small Aδ- and C-fibers. It is anticipated that due to an overall increase of survival, the prevalence of CIPN will increase in the next decade [10].

Paclitaxel is an antineoplastic agent most used in ovarian, breast, and prostate cancer treatment. Symptoms such as pain and numbness in hands and feet are predominately sensory due to paclitaxel accumulation in the dorsal root ganglia (DRG). The DRGs mainly consist of sensory neuron cells and are highly susceptible to paclitaxel accumulation due to a more permeable blood-nerve barrier [11].

Paclitaxel induces axonal transport disruption via microtubule stabilization, changes in morphology and function of mitochondria, and inflammation. These pathological changes cause symmetrical damage of axons and nerve fiber loss.

With the occurrence of paclitaxel-induced peripheral neuropathy, the treatment schedule of patients usually needs to be altered—e.g., via dosage reduction or treatment stop—to prevent further progress of neuropathic symptoms.

In the following section, we review the antineoplastic mechanism of action of paclitaxel and its interference with cells of the peripheral nervous system via alteration of microtubule dynamics, mitochondria changes, induction of oxidative stress, and inflammation.

## 2. Paclitaxel

Paclitaxel was first extracted and isolated from the bark of the Pacific yew tree (*Taxus brevifolia*) in 1971 [12]. It became more viable with semi-synthetical production from a paclitaxel precursor from the European yew tree’s needles.

Currently, paclitaxel is one of the most used taxanes, besides other formulations such as docetaxel, cabazitaxel, and nab-paclitaxel. Taxanes are commonly used to treat ovarian, breast, small- and non-small-cell lung, prostate, stomach, esophageal, bladder, pancreas head and neck cancer, as well as Kaposi’s sarcoma and melanoma [13,14,15,16].

Structurally paclitaxel is a diterpenoid pseudoalkaloid with a taxane ring as its nucleus (C_47_H_51_NO_14_). Due to its hydrophobic properties, a vehicle must be used for proper administration. Usually, a 50/50 solution of dehydrated ethanol and a polyethoxylated castor oil, Kolliphor EL, is used [17,18]. Kolliphor EL, formerly known as Cremophor EL, can form micelles around paclitaxel and thus keep paclitaxel in solution. However, the Kolliphor EL solution has several disadvantages. For once, it can alter the disposition and pharmacokinetics of paclitaxel [19]. Further, it can induce acute hypersensitivity reactions with symptoms of dyspnea, flushing, rash, chest pain, tachycardia, hypotension, angioedema, and generalized urticaria [20]. To prevent these possible side effects, patients need to be pre-treated with antihistamines and corticoids [21]. Moreover, Kolliphor EL can have direct effects on the peripheral nervous system (PNS) as well. It is neurotoxic itself due to the induction of axonal swelling and degeneration of DRG neurons and vesicles [22].

There is another paclitaxel formulation, nanoparticle albumin-bound paclitaxel (nab-paclitaxel), which is commonly used. Like CreEL-paclitaxel, nab-paclitaxel mainly accumulates in neurofilament 200-positive large-caliber neurons and less in Isolectin B4-, or calcitonin gene-related peptide-positive small-caliber neurons. Sensory nerve conduction studies demonstrated altered sensory dysfunction between the two formulations. It indicates that different “carriers” may impact the severity of neuropathy induced by paclitaxel via different tissue uptake [23].

## 3. Antineoplastic Mechanism of Paclitaxel

Paclitaxel and other taxanes can impair the increased abnormal cell proliferation and mitosis rate of tumor cells. During mitosis, microtubules are essential players in the segregation process of chromosomes into the daughter cells [24]. Especially during the interphase, microtubules act as tracks for organelles and the nucleus [25].

Microtubules are formed by α- and β-tubulin heterodimers to act as intracellular dynamic cytoskeletal polymers. Their dynamic stems from the ability to polymerize and depolymerize. Polymerization can also be described as rapid growth, and depolymerization can be characterized as shrinkage [26]. Due to these properties, the cell can rapidly reorganize its cytoskeleton. Both α- and β-tubulin are bound to guanosine triphosphate (GTP).

GTP-bound tubulin units are incorporated at the microtubule ends during the polymerization. This process makes the microtubule grow and form a stabilizing cap. Afterward, GTP gets hydrolyzed into GDP. This hydrolyzation process releases energy and destabilizes the microtubules and the microtubules splay apart at the end [25,27].

**Figure 1:**
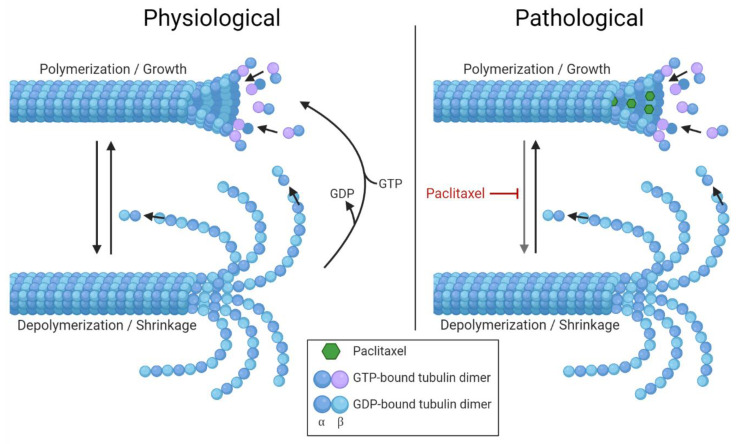
Paclitaxel enhances microtubule stability. Under physiological conditions, guanosine triphosphate (GTP)-bound tubulin dimers get incorporated at the growing end of the microtubules. This structure is supposed to form a stabilizing cap of GTP-bound tubulin. The conformation change in tubulin dimers is due to the GTP getting hydrolyzed into guanosine diphosphate (GDP), and this destabilizes the microtubule lattice. Through the loss of the GTP-tubulin cap, the microtubules are getting depolymerized. This process of microtubule depolymerization gets prevented by paclitaxel binding to β-tubulin (pathological condition). Created with BioRender.com (accessed on 20 September 2021).

Paclitaxel is able to interact with β-tubulin, which interferes with the dynamic process [28]. Through small openings in the microtubule lattice, paclitaxel can enter and bind to β-tubulin [29,30]. This causes a strengthening between the tubulin subunit’s lateral contacts [31]. Hence, the depolymerization is suppressed and the microtubules are stabilized (Figure 1).

Due to the interference with paclitaxel, mitosis is arrested between the metaphase and anaphase called the G_2_/M phase [17]. This leads to membrane potential reduction in mitochondria and causes the opening of the permeability transition pore channel. Proapoptotic factors release subsequently induces apoptosis [32]. Moreover, antiapoptotic effects are impaired, as paclitaxel is able to bind directly to B-cell lymphoma 2 (BCL2) [33].

## 4. Paclitaxel-Induced Peripheral Neuropathy

Paclitaxel-induced peripheral neuropathy is associated with a length-dependent axonal sensory neuropathy [34]. The induced neurotoxicity depends on paclitaxel dosage and infusion time and can be fostered by underlying conditions or co-treatment with other drugs [35,36]. This makes the severity and occurrence of paclitaxel-induced peripheral neuropathy hard to predict. First symptoms such as numbness, tingling, and/or allodynia in the patient’s fingers and toes can be observed 24 to 72 h post-injection. Numbness and tingling can reach up to the lower leg and wrists of patients in a "glove and stocking" like manner [37,38]. These symptoms occur in up to 97% of all treated patients, especially when the cumulative dose exceeds 1400 mg/m^2^ [39,40]. A total of 60% of all treated patients manifest chronic paclitaxel-induced peripheral neuropathy [40].

Due to the microtubule-stabilizing properties of paclitaxel, it damages peripheral axons symmetrically, and described symptoms are the phenotypic correlations of an axonal dying back pattern. In severe cases, axonal degeneration occurs along with secondary demyelination [41]. Axonal injury can be seen in nerve conduction studies by a reduction or a complete loss of sensory nerve action potentials [42]. Paclitaxel-induced impairment is most prominent in large myelinated Aβ-fibers [35,37]. This is reflected by symptoms such as the impaired sensation of vibration or touch. Further, a high concentration of paclitaxel leads to a loss of intraepidermal nerve fibers (IENFs). These nerve fibers enter the epidermis as Aδ- and C-fibers. Loss of those fibers is reflected in taxol-induced hyperalgesia and pain sensation in patients. In comparison, treatment with lower doses of paclitaxel induces an increase in IENFs diameter and a degeneration of terminal arbor [43]. This overall leads to a nerve fiber loss in the epidermis, manifesting in symptoms of thermal hyperalgesia and mechanical allodynia [44].

An important molecule that executes axonal degeneration is SARM1 (Sterile Alpha And TIR Motif Containing 1). It is activated in demyelinated axons and its NADase, which downstream can initiate the process of axonal self-destruction [45]. A class of isoquinoline small molecules has been described to reversible inhibit SARM1 NADase leading to protection against traumatic injuries and mitochondrial damage [46]. It has been recently described that these molecules were also able to prevent the loss of IENFs and partially protect axonal functions in a paclitaxel-induced peripheral neuropathy animal model [47].

## 5. Mechanisms of Neuronal Injury and Neuronal Dysfunction

### 5.1. Altered Microtubules Dynamics

Paclitaxel is a highly effective chemotherapeutic agent due to the stabilization of microtubules in cancer cells. However, this stabilization also does affect microtubules of sensory neurons in the DRG and axons of the PNS. Over the last decades, a number of in vitro and in vivo models for paclitaxel-induced neuropathy have been established (reviewed in [48,49]). There is evidence that paclitaxel impairs the transport of proteins, organelles, nutrients, neurotransmitters, and mRNA [50,51,52,53,54]. Downstream of impaired mitochondrial transport lay the undersupply of ATP, which is needed for axonal transport. This way, axonal transport is further decreased. Further, impaired mitochondrial transport promotes the breakdown of the ion gradient in the axolemma, which is crucial for electrochemical impulses [55,56]. Hence, microtubule stabilization leads to loss of axonal transport, which promotes axonal degeneration or axonopathy and ends in peripheral neuropathy [57]. Moreover, the impairment of axonal trafficking of RNA transport granules inhibits bclw translation, which reduces the expression of Bcl2 family member Bclw (Bcl2l2). The Bcl-2 homology (BH)4 domain of Bclw is able to bind inositol 1,4,5-trisphosphate receptor (IP_3_R)1. IP_3_R lays upstream of axonal degradation via the release of cytoplasmic calcium, leading to increased calcium flux into mitochondria and the proteolysis of calpain, a calcium-dependent enzyme [58]. Due to the impairment of axonal trafficking during paclitaxel treatment, IP_3_R is not bound and able to induce axonal degeneration [59].

### 5.2. Mitochondria and Oxidative Stress

Paclitaxel treatment impairs not only the axonal transport of mitochondria but also their morphology and function. Mitochondria of myelinated fibers and unmyelinated C-fibers and paclitaxel treatment swell up and vacuolize with the fragmentation of cristae due to the opening of the mitochondrial permeability transition pore [50,60,61]. These changes correlate with the pain-like behavior in rats [60]. Furthermore, the paclitaxel-induced alterations in the permeability transition pore opening also cause changes in calcium flow. Calcium flow changes induce deficiencies in the mitochondrial respiratory chain, which leads to ATP deficits. These ATP deficits are detectable during paclitaxel-induced pain sensation and persist even after the peak of pain sensation [62]. It is thought that impairment of mitochondrial function moreover results in degeneration of terminal arbors and further fostering the generation of neuropathic pain [63].

Furthermore, the response to oxidative stress is impaired by paclitaxel treatment, further increasing the ATP deficit [64]. It was observed that co-treatment with antioxidants reduced mitochondrial dysfunction, intraepidermal nerve fiber loss, and pain [65,66]. Mitochondrial damage and reactive oxygen species (ROS) are closely dependent since mitochondrial damage cause the production of ROS, i.e., H_2_O_2_ formation. ROS, in turn, cause damage to the mitochondria, inducing DNA fragmentation and mitochondrial membrane potential loss [67]. Hence, ROS are crucial players in the oxidative stress reaction and are increasingly expressed after paclitaxel treatment [68]. Along with elevated ROS levels, manganese activity, copper-zinc SOD activity, and glutathione (GSH) peroxidase antioxidant enzyme activities are induced by paclitaxel [68]. Notably, Fidanboylu and colleagues could demonstrate that a non-specific ROS scavenger, N-tert-Butyl-α-phenylnitrone (PBN), could prevent the development of paclitaxel-induced peripheral neuropathy [69]. In chronic myelogenous leukemia K562 cells, apoptosis was induced with the generation of ROS and GSH depletion. Taxol further caused the increase of activity of c-Jun NH2-terminal kinase (JNK) and p38, which are known mediators of the stress activation pathways [70].

There is also evidence that epidermal matrix-metalloproteinase 13 (MMP-13) promotes degeneration of unmyelinated nerve fibers following paclitaxel treatment. In zebrafishes that are exposed to paclitaxel, H_2_O_2_ reactive species are increased in basal keratinocytes, originating from damaged mitochondria. This upregulates MMP-13, which contributes to matrix degradation and degeneration of axons in the epidermis [71]. While MMP-13 inhibition did not alter mitochondrial damage, it was able to prevent axon degeneration [72]. In general, matrix-metalloproteinases (MMPs) are regulated by ROS: expression of MMP-2 has been increased in a breast cancer cell line after being exposed to a mitochondrial ROS inducer [73]. In prostate cancer cells, increased H_2_O_2_ upregulated MMP-3 expression by inhibiting the MMP-3 suppressor [74]. Moreover, MMP-3 has been linked to macrophage accumulation in the DRGs.

### 5.3. Inflammation and Pain

Paclitaxel induces inflammation by cytokine and chemokine release and infiltration of non-resident macrophages into the DRGs, and this inflammatory reaction results in neuropathic pain [75]. Most prevalent are increased interleukin-1β (IL-1β), IL-8, and tumor necrosis factor α (TNF-α) expression [76]. An anti-TNF-α agent or an IL-1 receptor agonist was able to prevent paclitaxel-induced pain [77]. The same goes for IL-8 and its receptors, inhibition of those reduced nociception as well as mechanical and cold hypersensitivity [78].

Chemokine receptors that are upregulated in the DRG after paclitaxel treatment are C-X-C chemokine receptor type 4 (CXCR4) and receptor for advanced glycation end products (RAGE). The increased expression of those receptors goes along with an increased macrophage accumulation in the sciatic nerve. Interestingly paclitaxel-induced allodynia could be prevented with CXCR4 and RAGE antagonists [79].

Further, chemokines C-X-C motif chemokine ligand 1 (CXCL1), C-X-C motif chemokine ligand 12 (CXCL12), and C-X3-C motif chemokine ligand 1 (CX3CL1) are reported to be involved in the immune reaction following paclitaxel exposure. On the one hand, inhibition of CXCL1 was able to reverse paclitaxel-induced mechanical allodynia [80]. On the other hand, CXCL1’s receptor CXCR2 and PI3Kγ have been found to be upregulated after paclitaxel treatment and may contribute to mechanical hypersensitivity [81]. CXCL12 expression is increased after paclitaxel treatment and correlates with increased excitatory postsynaptic currents in the spinal dorsal horn neurons. CXCL12 could induce mechanical allodynia since inhibition of the CXCL12 signaling pathway improved paclitaxel-induced mechanical allodynia [82]. Lastly, paclitaxel induces an upregulated expression of CX3CL1 in spinal neurons. Inhibition of this chemokine was reported to reduce macrophage-neuron interactions [83]. CX3CL1 possibly lies downstream of transcriptional factor NF-κB activation and histone acetylation. At the same time, NF-κB itself is a key player in thermal hypersensitivity in paclitaxel-induced peripheral neuropathy [84].

As another part of the innate immune system, the complement system was linked via complement component 3 (C3) activation to paclitaxel-induced peripheral neuropathy. In vitro, paclitaxel enhanced C3 activation, and in vivo knock-out of C3 ameliorated paclitaxel-induced touch sensitivity and increased intradermal nerve fibers [82].

In recent years, cannabinoid receptors 1 (CB1) and 2 (CB2) were discovered to possibly play a role in the immune reaction, microglia activation, and pain sensation after paclitaxel treatment. Increased expression of CB2 alongside chemokine (C-C motif) ligand 2 (CCL2) and IL-6, IL-4, and IL-10 expression after paclitaxel treatment is associated with a dysregulation of microglia in the dorsal horn [85,86,87]. CB1 and CB2 agonists inhibited spinal glial activation and IL-1β, IL-6, TNF-α, and CCL2 up-regulation. Further, they also modulated spinal p38 MAPK and NF-κB activation [88,89,90]. These effects prevented cold and mechanical allodynia [89]. These effects almost mirror co-treatment with minocycline. In this case, cannabinoid receptor agonism could have analgesic effects and, in return, reduce paclitaxel-induced neuropathic pain [86,88].

Besides paclitaxel-induced microglial activation in the dorsal horn, an astrocyte activation, independent from microglia activation, in the spinal cord is detectable [91].

The main players of inflammation in the DRG are accumulated macrophages. Initially, an upregulation of MMP-3 after paclitaxel exposure is detected. MMP-3 can break down the extracellular matrix and attract macrophages. This is followed by an upregulation of CD163, a macrophage marker, and C11b, a monocyte and macrophage marker. CD11b is further implied in pathogen recognition, phagocytosis, and cell survival. The accumulated macrophages in the DRG express pro-inflammatory markers desensitizing primary sensory afferent resulting in neuronal and glial damage. It is most likely that altered neuronal and glial physiology disrupts spinal dorsal horn input and generates neuropathic pain [92,93,94,95]. Another macrophage attracting chemokine, monocyte chemoattractant protein 1 (MCP-1), is increasingly expressed in small nociceptive DRG neurons as well as spinal astrocytes of paclitaxel treated rats [96]. Increased MCP-1 expression leads to an upregulation of its cognate receptor, C-C chemokine receptor type 2 (CCR2), in large and medium-sized myelinated neurons. While increased overexpression of CCR2 induced increased calcium spikes in CCR2-positive neurons, macrophage depletion prevented increased CCR2 expression and reversed intra-epidermal nerve fiber loss and mechanical hypersensitivity [75]. Moreover, activation of the Toll-like receptor 4 (TLR4) could go hand in hand with increased MCP-1 expression [97,98].

The activation of spinal astrocytes and satellite glial cells (SGC) is thought to be a secondary response to macrophage activation and neuronal damage. Paclitaxel induced an activation state in SGC and Schwann cells detectable in an upregulation of the transcription factor 3 (ATF3) [91]. Further, paclitaxel treatment leads to increased gap junction coupling between the SGC, promoting ATP and neurotransmitter expression by the SGC. While SGC are essential players in neuronal homeostasis, increased ATP and neurotransmitter expression paradoxically could induce neuronal death [99]. Furthermore, the previously described increased expression of TLR4 could navigate TNF-α release of SGC and thus inflammation, pain sensation, and transient receptor potential (TRP) channel activation [68,100,101,102]. For example, TRPA1 and TRPV4 have already been implicated to be key roles in the induction of paclitaxel-induced cold hypersensitivity [103].

### 5.4. Drug Transporters Involvement in Paclitaxel Translocation

In the last few years, organic-anion-transporting polypeptides (i.e., OATP1B1 and OATP1B3) are increasingly recognized to be important molecules for translocation of paclitaxel across the plasma membrane. While hepatocytes are known to use OATPs to translocate paclitaxel, their expression and role in the peripheral nervous system are not well known. Recently, it has been shown that OATP1B2, the mouse homolog to OATP1B1 and OATP1B3, is expressed in mouse DRG and that pharmacological inhibition and knock-out of OATP1B2 ameliorated paclitaxel-induced peripheral neuropathy [104]. This indicated that OATP1B2 could be a driving force of paclitaxel accumulation in the DRG neurons. Moreover, OATP1B3 has been found to be expressed on ovarian and associated cancer cell lines [105], colorectal and pancreatic cancer cells [106], and castration-resistant prostate cancer cells [107]. OATP1B1 expression was confirmed on ovarian cancer cells [105].

Another candidate for paclitaxel transport across the cell membrane could be organic anion transporter 2 (OAT2). Interactions between OAT2 and paclitaxel have been observed [108], further underlined by increased accumulation of radiolabeled paclitaxel in OAT2 expressing oocytes [109]. However, OAT2 has three transcript variants and the corresponding variant has not been identified yet. OAT2 transcript variant 2 was able to translocate paclitaxel but is not expressed on the examined cell lines, while OAT2 transcript variant 1 also had paclitaxel as its substrate in disregard on which cell type it is expressed [110].

Overall, it is crucial to understand the accumulation mechanism of paclitaxel in the sensory neuron, which could be achieved with further studies of drug transporters. Understanding and manipulating the transport process could prevent or ameliorate paclitaxel-induced peripheral neuropathy.

## 6. Summary

**Figure 2:**
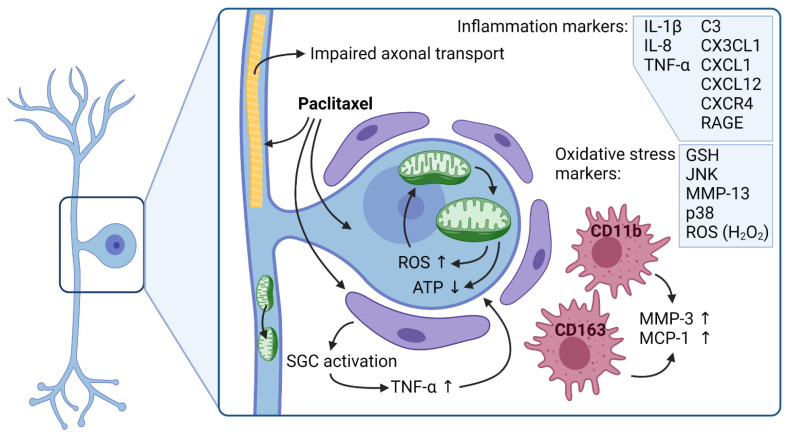
Pathomechanisms of paclitaxel-induced peripheral neuropathy in the peripheral nervous system. ATP—adenosine triphosphate, C3—complement component 3, CD11b—cluster of differentiation 11B, CD163—cluster of differentiation 163, CX3CL1—C-X3-C motif ligand 1, CXCL1—C-X-C motif chemokine ligand 1, CXCL12—C-X-C motif chemokine ligand 12, CXCR4—as C-X-C chemokine receptor type 4, GSH—glutathione, IL—interleukin, JNK—c-Jun N-terminal kinase, MCP-1—monocyte chemoattractant protein 1, MMP—matrix-metalloproteinase, RAGE—receptor for advanced glycation end products, ROS—reactive oxygen species, SGC—satellite glial cell, TNF-α—tumor necrosis factor; Created with BioRender.com (accessed on 20 September 2021).

**Figure 3:**
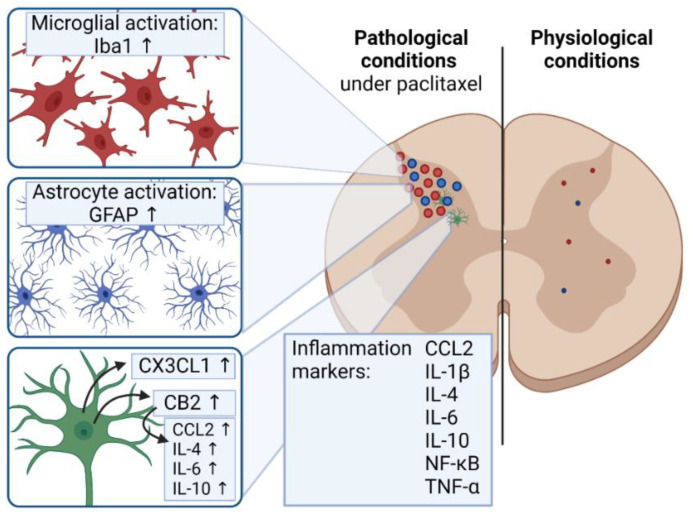
Pathomechanisms of paclitaxel-induced peripheral neuropathy in the spinal cord. CB2—cannabinoid receptor type, CCL2—C-C motif ligand 2, CX3CL1—C-X3-C motif ligand 1, GFAP—glial fibrillary acidic protein, Iba1—ionized calcium-binding adapter molecule 1, IL—interleukin, NF-κB—nuclear factor kappa B, TNF-α—tumor necrosis factor; Created with BioRender.com (accessed on 20 September 2021).

Paclitaxel is a potent antineoplastic agent that in tumor cells induces cell death via microtubule stabilization. However, it can cause chemotherapy-induced peripheral neuropathy in up to 97% of all treated patients. Typically, paclitaxel induces a length-dependent axonal sensory neuropathy correlating with the dose, infusion time, underlying conditions, and co-treatment with other drugs. Most prevalent are the symptoms of numbness and tingling in the feet and hands of the patients. While it acts as a microtubule-stabilizing and apoptosis-inducing agent in cancer cells, these properties can symmetrically damage peripheral axons and induce an axonal dying back pattern, mainly affecting Aβ-fibers. The microtubule stabilization impairs the transport of proteins, organelles, nutrients, neurotransmitters, and mRNA in the PNS. ATP undersupply and paclitaxel-induced morphological changes of mitochondria correlate with increased pain sensation. Typical inflammation markers in the PNS after paclitaxel treatment are IL-1β, IL-8, and TNF-α, which are thought to elicit pain sensation. Other markers involved in the immune response include CXCR4, RAGE, CXCL1, CXCL12, CX3CL1, and C3 (Figure 2). Interestingly, cannabinoid receptor agonists could ameliorate paclitaxel-induced neuropathic pain through the involvement in the immune reaction. Microglia and astrocytes independent from each other mainly get activated in the spinal cord (Figure 3). At the same time, SGC are the glial cells activated in the PNS, which in return can increasingly express TNF-α and neurotransmitters promoting neuronal death. Moreover, CD11b and CD163 positive macrophages infiltrate the DRG attracted by MMP-3 and MCP-1, which further can induce neuronal degeneration.

Especially the immune response modulation in in vivo studies with rodents were able to ameliorate or prevent paclitaxel-induced peripheral neuropathy. However, with prolonged survival rates of cancer patients, paclitaxel-induced peripheral neuropathy becomes an increasingly larger problem. Still, thus far, no approaches are available that have reached clinical use to prevent or decrease neuropathy severity.

## Data Availability

Not applicable.
